# Precision medicine meets the DNA damage response in pancreatic cancer

**DOI:** 10.18632/oncoscience.392

**Published:** 2018-02-22

**Authors:** Lukas Perkhofer, Anett Illing, Johann Gout, Pierre-Olivier Frappart, Alexander Kleger

**Affiliations:** Department of Internal Medicine I, University Hospital Ulm, Ulm 89081, Germany

**Keywords:** pancreatic cancer, DNA damage repair, ATM, therapy

Pancreatic Ductal Adenocarcinoma (PDAC) is one of the major cancer problems in the present but even more in the future. Today, over 7% of all cancer deaths in the USA are PDAC caused and the incidence is predicted to increase further over the next decade (reviewed in [1]). The step wise progression from ductal metaplasia (ADM) via acinar to ductal reprogramming steps (ADR) to pancreatic intraepithelial neoplasia (PanIN) and finally frank PDAC is orchestrated through an interplay of various mutations. Recent genome sequencing studies have shed light on the mutational landscape of PDAC including a small set of key driver mutations like KRAS, TP53, CDKN2A, or SMAD4 guided by a high number of passenger mutations. Thereby, the latter establish the characteristic intra- and intertumoral heterogeneity [2] but also allows for the first time practical PDAC subtyping. Here, the so-called “unstable PDAC” subtype comprises a relevant and maybe best druggable new entity [3]. Typically, the unstable PDAC subtype harbours mutations in genes involved in DNA damage response (DDR) such as *BRCA1/2, PALPB2* and *ATM* [2], which are associated with increased chemoresistance, aggressive disease course and thus dismal prognosis. Specifically, BRCA1/2-mutations in PDAC seem to generate a tumour biology being more sensitive to platinum-based chemotherapies and PARP inhibition [3]. However, genotype-tailored therapies for non-BRCA1/2-mutated unstable PDACs, such as mutations in the serine/threonine protein kinase Ataxia-telangiectasia-mutated (ATM), remain to be identified.

The current palliative standard of care in PDAC therapies is still based on a combination of various conventional chemotherapeutic agents exemplified in the highly potent FOLFORINOX regimen (5-FU, leucovorine, oxaliplatine and irinotecan). Albeit promising this regimen remains far away from a “one-size-fits-all-approach” as the clinical response usually has a broad range with rare long-term survivors. This range appears to be defined by the complex PDAC genetic heterogeneity, where distinct driver and passenger mutations prevent the aimed universal treatment approach as illustrated by a myriad of failed trials [1]. Thus, understanding PDAC biology with respect to the cancerous mutational make-up will help to develop druggable targets and DDR genes might the most promising targets at this stage.

**Figure 1:**
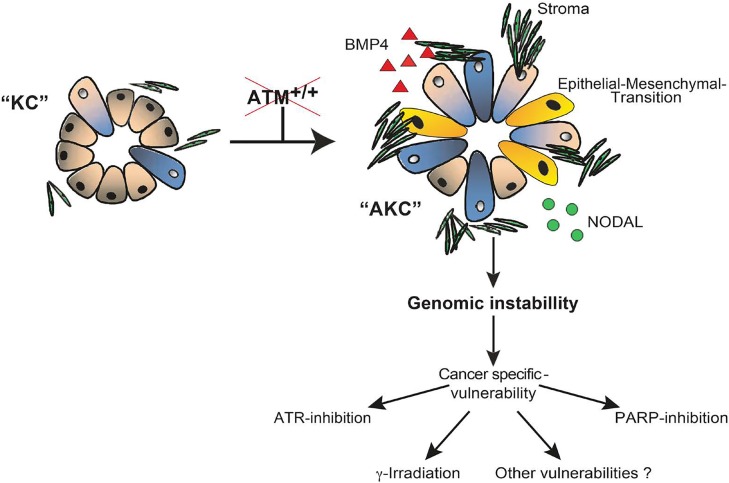
The loss of Atm (“AKC”) drives progressive alterations in a p48-Cre//KrasG12D (“KC”) background AKC driven PDAC is stroma enriched and shows hyperactivation of the Nodal-Smad 1/3 and Bmp-Smad 2/3 axis. Epithelial-Mesenchymal-Transition is enriched with a specific EMT signature linked to the loss of Atm. AKC tumors were found to be highly genomic instable, resulting in a specific treatment vulnerability to ATR and PARP-inhibition as well as irradiation.

ATM has a major role in the DNA-damage response (DDR). It phosphorylates key mediators in cell cycle arrest, DNA repair, apoptosis and senescence [4, 5]. Recent combined large-scale sequencing studies reported *ATM* mutations in up to 18% of human PDAC patients [5, 6]. To delineate the impact of ATM-deficiency on pancreatic carcinogenesis, we established a genetic mouse model expressing KrASG12D and lacking ATM specifically in the pancreas, the “AKC” mouse. The AKC-mouse develops parenchymal foci with partially disrupted acinar tissue already at an early stage of 5 weeks of age, and at 10 weeks-old it shows strong ADR. In line, this occurs with pronounced SOX9 expression, hyperproliferation and stromal infiltration compared to ATM-expressing KRASG12D (“KC”) mice. Accelerated dysplastic growth in the pancreas was driven by increased TGFB superfamily signalling including a hyperactive Nodal-Smad1/3 and Bmp-Smad2/4 axis [5]. Moreover, molecular depletion of ATM resulted in increased epithelial-to-mesenchymal transition (EMT), especially in ADM-regions, and an EMT specific gene expression signature became evident (see Figure 1). In line, AKC-mice suffered from a six-fold increase of liver metastasis. Thus, deletion of the DDR gene *ATM* in PDAC dramatically perturbs pancreatic biology generating a certainly more aggressive but eventually also more vulnerable subtype [5].

Altered DNA repair is a hallmark of cancer that results in genomic instability and accumulation of genetic changes [7]. Interestingly, mutations in DNA repair genes may sensitize to innovative treatments e.g. by inducing synthetic lethality due to the inhibition of complementary DNA repair mechanisms. We recently applied such an approach in our AKC mouse model for pancreatic cancer [8]. We observed that AKC-mice faithfully reflect the genomic instable PDAC subtype and thereby can be used as platform to further validate specific therapies [8]. In doing so, we increased the load of DNA damage in AKC tumour cells to interrogate with the remaining DNA repair capacity or by increasing replicative stress using various inhibitors: PARP1 was first targeted as a key player in base excision repair and alternative non-homologous end joining. Application of olaparib, a PARP inhibitor, strongly induced apoptosis in AKC cells due to increased double-strand breaks (DSB), while ATM proficient lines remained virtually unaffected. This effect could be considerably potentiated by the combination of PARP-inhibitors with gemcitabine *in vitro* and *in vivo*. Furthermore, low dose γ-irradiation in the context of olaparib-treatment even allowed dose-saving (see Figure 1) [8]. The inhibition of alternate DDR signalling routes in our PDAC model also indicated compensational signalling for the ATM loss by both ATR and PRKDC (DNA-dependent protein kinase). In line with this notion, ATR inhibition again potentiated gemcitabine activity and thereby slowed down tumour growth of AKC-cells *in vivo* [8]. Albeit ATM-deficient PDAC seems to be highly sensitive to PARP- and ATR-inhibition, prolonged treatment bears the risk of resistance and strategies to overcome this are still missing [8]. One solution could be the identification of other synthetic lethality or synergistically interacting pathways in the context of ATM-deficiency in PDAC. Based on the acquired data the combination of ATR- and PARP-inhibition maybe potent to overcome resistance in an ATM-deficient background [8].

Generally, the treatment strategies in the different PDAC subtypes have to be individualized and the backbone chemotherapy challenged in light of the mutagenome as well. Defects in DDR genes such as ATM might be the most promising and best understood druggable vulnerabilities in cancer at the current state. However, various unknowns remain in light of ATM-deficiency and thus prevent further personalized steps in DDR defective unstable PDAC. To bring such therapies to the clinic, the consequences of individual DDR gene mutation on PDAC biology needs to be thoroughly investigated and patients have to be screened, as we regularly do for *KRAS*, to substantiate our preclinical data in the warranted randomized controlled trials. All these novel approaches may contribute to finally use the back door in unstable PDAC provided by DDR gene mutations to specifically and efficiently target them and therefore significantly improve the prognosis of those patients.
